# Artificial urinary sphincter revision for urethral atrophy: comparing single cuff downsizing and tandem cuff placement

**DOI:** 10.1590/S1677-5538.IBJU.2016.0240

**Published:** 2017

**Authors:** Brian J. Linder, Boyd R. Viers, Matthew J. Ziegelmann, Marcelino E. Rivera, Daniel S. Elliott

**Affiliations:** 1 Department of Urology, Mayo Clinic, Rochester, MN, USA

**Keywords:** Urinary Sphincter, Artificial, Urinary Incontinence, Male

## Abstract

**Objective:**

To compare outcomes for single urethral cuff downsizing versus tandem cuff placement during artificial urinary sphincter (AUS) revision for urethral atrophy.

**Materials and Methods:**

We identified 1778 AUS surgeries performed at our institution from 1990-2014. Of these, 406 were first AUS revisions, including 69 revisions for urethral atrophy. Multiple clinical and surgical variables were evaluated for potential association with device outcomes following revision, including surgical revision strategy (downsizing a single urethral cuff versus placing tandem urethral cuffs).

**Results:**

Of the 69 revision surgeries for urethral atrophy at our institution, 56 (82%) were tandem cuff placements, 12 (18%) were single cuff downsizings and one was relocation of a single cuff. When comparing tandem cuff placements and single cuff downsizings, the cohorts were similar with regard to age (p=0.98), body-mass index (p=0.95), prior pelvic radiation exposure (p=0.73) and length of follow-up (p=0.12). Notably, there was no difference in 3-year overall device survival compared between single cuff and tandem cuff revisions (60% versus 76%, p=0.94). Likewise, no significant difference was identified for tandem cuff placement (ref. single cuff) when evaluating the risk of any tertiary surgery (HR 0.95, 95% CI 0.32-4.12, p=0.94) or urethral erosion/device infection following revision (HR 0.79, 95% CI 0.20-5.22, p=0.77).

**Conclusions:**

There was no significant difference in overall device survival in patients undergoing single cuff downsizing or tandem cuff placement during AUS revision for urethral atrophy.

## INTRODUCTION

While the use of an artificial urinary sphincter (AUS) in the management of severe male stress urinary incontinence has been associated with excellent long-term outcomes, many patients will experience recurrent incontinence (1-3). Notably, in several large series, urethral atrophy has been reported as the most common cause for non-mechanical failure or device revision (4, 5). It has been hypothesized that in this setting urethral atrophy occurs because the AUS achieves continence by applying constant circumferential compression of the corpus spongiosum, which over time leads to tissue atrophy (6).

Notably, several surgical options for AUS revision in cases of urethral atrophy have been reported, including: changing the location of the urethral cuff (7), downsizing the single urethral cuff (8), placement of a second (tandem) urethral cuff (9, 10), transcorporal cuff placement (11, 12) or revising the pressure-regulating balloon (5). Unfortunately, there is a paucity of data comparing these management strategies, and those currently available are not in the setting of AUS revision for urethral atrophy (13). Given the lack of available data, the choice between these management options is based on the local tissue quality, location of the in-situ urethral cuff and surgeon preference.

Thus, we sought to compare outcomes for single urethral cuff downsizing versus tandem cuff placement during AUS revision for urethral atrophy.

## MATERIALS AND METHODS

After obtaining Institutional Review Board approval, we identified 1778 AUS surgeries at our institution from June 1^st^ 1990 to December 31^st^ 2014. Of those patients, 406 were the initial AUS revisions (i.e. secondary surgery), including 69 for urethral atrophy. Patients were excluded from analysis if they underwent revision surgery at another institution, underwent primary AUS placement secondary to neurogenic bladder dysfunction, were less than 18 years old at the time of primary AUS placement, or declined research consent. Both the primary implantation and revision surgery were performed at our institution in all cases. All implanted AUS devices were American Medical Systems 800 (AMS 800; American Medical Systems, Inc., Minnetonka, Minnesota, USA). The revision surgeries were performed by three surgeons.

With regard to our approach to evaluation of recurrent stress urinary incontinence after AUS placement, we typically obtain a history and physical, cystoscopy, and x-ray imaging (as contrast is instilled at the time of surgery in our primary placements). The diagnosis of urethral atrophy is confirmed during cystoscopy, when incomplete urethral coaptation is visualized with device cycling (with adequate fluid in the system on radiographic imaging). Patients confirmed to have urethral atrophy are considered for surgical AUS revision depending on symptom severity, patient preferences and comorbidities.

**Figure 1:**
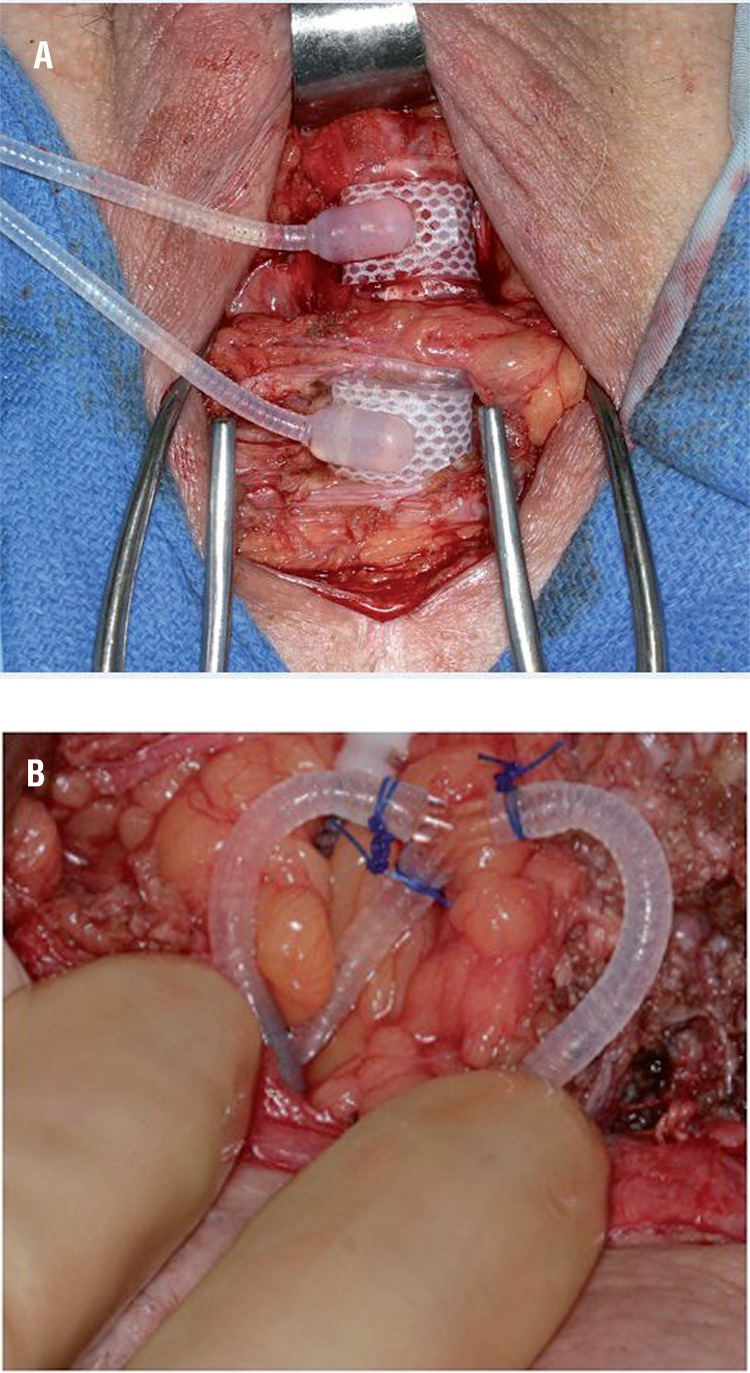
Tandem urethral cuff placement (a), connection of tandem urethral cuff to in-situ system (b).

The decision to proceed with single cuff downsizing versus tandem cuff placement was at the discretion of the treating surgeon. Our tandem cuff placements are performed with the second cuff placed roughly 1-2cm distal to the primary cuff (Figure-1a). Additionally, we use a Y-shaped adapter, secured with free ties of non-absorbable suture to connect the pump tubing to both cuffs (Figure-1b). Furthermore, we add 1-3cc of fluid to the system to account for the additional volume sequestered in the cuff. In cases of severely atrophic urethral tissues (measurement <3.5cm), distal urethral tapering or difficult dissection planes (e.g. in some cases with prior pelvic radiation therapy or urethral sling placement), we utilize a transcorporal approach, as previously described (11, 12). As all of the initial primary AUS placements in this study were performed at our institution, we are typically unable to relocate the cuff more proximally during subsequent revision surgery, as the primary cuff is placed as proximal as possible. Notably, we perform primary implantations with the cuff placed circumferentially around the bulbospongiosus muscle.

Individual charts were reviewed to evaluate pertinent clinical and surgical comorbidities, details of both the primary and secondary devices, primary device outcome including time to failure, revision management strategy (single cuff versus tandem cuff), and secondary device outcome. Given the retrospective study design patients, we did not have standardized follow-up. Instead, following device placement, patients are evaluated six weeks post-operatively for device activation. Following this, patients are followed via office evaluation on an as needed basis. Additionally, the Mayo Clinic AUS Registry monitors outcomes periodically by correspondence to the patient. Details regarding device survival were obtained from last office examination, subsequent operative report, written or telephone correspondence.

Statistical analysis was performed using the JMP 11 software package (SAS Institute, Inc.: Cary, NC). Patients were divided into cohorts based on management strategy, that is tandem cuff placements (including tandem and tandem transcorporal cuff placements) or single cuff downsizing. Continuous features were summarized with medians and interquartile ranges (IQRs); categorical features were summarized with frequency counts and percentages. Device survival was estimated as time from AUS revision for urethral atrophy to subsequent repeat (tertiary) surgery (including explantation or device revision for any reason), or last known follow-up, using the Kaplan-Meier method and compared with the log-rank test. All statistical tests were 2-sided, with a p-value <0.05 considered statistically significant.

## RESULTS

**Figure 2:**
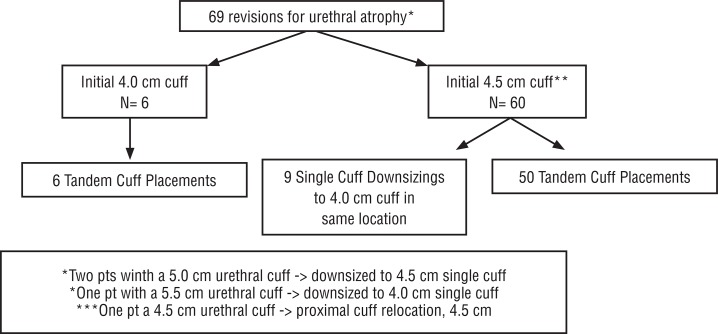
Distribution of cuff downsizings and tandem cuff placements by initial cuff size.

Of the 406 patients undergoing first time revision surgery during the study timeframe, 69 patients (17%) underwent revision surgery for stress urinary incontinence secondary to urethral atrophy. Three surgeons performed the revision cases; with regard to distribution, individually the surgeons performed 41 (59%), 18 (26%), and 10 (14%) procedures. The median time from primary AUS placement to revision for atrophy was 4.92 years (IQR 2.67, 7.79). Of the 69 revision surgeries for urethral atrophy at our institution, 56 procedures (82%) were tandem cuff placements, 12 (18%) were single cuff downsizings and in one case we were able to relocate the cuff proximally (single cuff). Of the 56 tandem cuff placements, 8 (14%) were performed with a transcorporal approach. The distribution of surgeries by initial cuff size is shown in Figure-2. Notably, during primary implantation 87% of patients (60/69) had a 4.5cm cuff placed, 8.7% (6/69) had 4.0cm cuff, 2.9% (2/69) had a 5.0cm cuff placed and 1.4% (1/69) had a 5.5cm urethral cuff placed. No patients underwent implantation of 3.5cm urethral cuff with either primary or revisions surgery.

The demographics of patients undergoing single cuff downsizing compared to those undergoing tandem cuff placement are shown in Table-1. Notably, the cohorts were similar with regard to age (p=0.84), body-mass index (p=0.99), prior pelvic radiation exposure (p=1.00) and time from primary surgery (p=0.53). There was no significant difference in the length of follow-up after device revision between those that underwent single cuff downsizing, compared to tandem cuff placement (median 1.3 years versus 2.24 years; p=0.28).

**Table 1:** Clinical and demographic features of patients undergoing Artificial Urinary Sphincter Revision for mechanical failure stratified by revision technique.

	Single Cuff Downsizing (n=12)	Tandem Cuff Placement (n=56)	p value
Age, years, median (IQR)	75.5 (71.8, 79.3)	74.6 (68.6, 80.6)	0.84
Body-mass index, kg/m^2^, median (IQR)	28.7 (26.1, 29.9)	27.5 (25.3, 30.9)	0.99
Prior pelvic radiation	4 (33.3%)	19 (33.9%)	1.00
Coronary artery disease	4/9 (44.4%)	9/35 (25.7%)	0.41
Time to primary failure, years, median (IQR)	6.13 (3.77, 8.46)	4.92 (2.54, 7.59)	0.53

Among patients undergoing revision for urethral atrophy (n=69), the median follow-up after revision surgery was 2.21 years (IQR 0.84, 6.76). During follow-up 19 patients underwent tertiary surgery, including 12 for device infection/urethral erosion, 3 for device malfunction and 4 for repeat urethral atrophy. All 4 cases of repeat urethral atrophy occurred in patients that underwent tandem cuff placement. Device infection or urethral erosion occurred in ten patients that underwent tandem cuff placement and two patients managed with cuff downsizing. Device malfunction occurred in two patients managed with tandem cuff placement and one treated with cuff downsizing.

**Figure 3:**
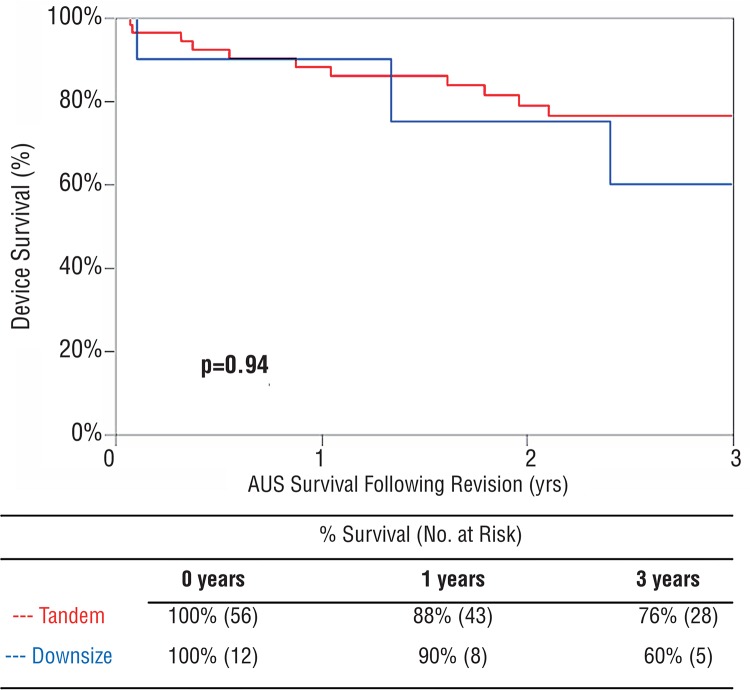
Overall device survival following revision surgery for patients with single cuff downsizing or tandem cuff placement.

There was no difference in 3-year overall device survival compared between single cuff and tandem cuff revisions (60% vs. 76%, p=0.94) (Figure-3). Likewise, there was no association of tandem cuff placement (ref. single cuff) and the risk of tertiary surgery for any cause (HR 0.95, 95% CI 0.32-4.12, p=0.94). Furthermore, there was no association of the risk of urethral erosion/device infection and tandem cuff placement (ref. single cuff) (HR 0.79, 95% CI 0.20-5.22, p=0.77). However, among those undergoing tandem urethral cuff placement, a transcorporal approach (8/56) was associated with adverse 3-year device survival compared to those placed without a transcorporal approach (44% vs. 80%, p=0.0016). Specifically, four patients that had transcorporal cuff placement had a repeat revision surgery, including three for device infection/erosion and one for device malfunction.

## DISCUSSION

We found here in a cohort of patients undergoing AUS revision for urethral atrophy, that there was not enough evidence to prove a difference in overall device survival in patients undergoing single cuff downsizing versus tandem cuff placement. Furthermore, there was no significant difference in the risk of urethral erosion/device infection between the two cohorts. These results augment the existing literature by providing comparative data on these management options in the setting of AUS revision for urethral atrophy.

Unfortunately, recurrent stress urinary incontinence following AUS implantation and subsequent revision surgery is not uncommon, impacting roughly 26% of patients undergoing primary AUS placement (1). Of these cases, a rate of revision surgery of 7.9% (range 1.9-28.6%) for urethral atrophy was reported in a pooled analysis (1). Notably, several surgical options for AUS revision in these cases have been reported, including: changing the location of the urethral cuff (7), downsizing the single urethral cuff (8), placement of a second (tandem) urethral cuff (9, 10), transcorporal cuff placement (11) or revising the pressure-regulating balloon (5). Notably, each series demonstrates excellent results for the technique proposed. For instance, in a study of 17 patients treated for recurrent incontinence with cuff downsizing over a seven-year period, Saffrian et al. found improvements in pad use (from 3.9/day to 0.5/day) and patient satisfaction (from 15% to 80%) with this technique (8). However, there is no comparative group against which to evaluate the results. As such, the evidence base that could be used to guide surgical management in cases of AUS revision for urethral atrophy is limited.

The one area with comparative evidence is the use of a single versus double/tandem urethral cuff placement, however this is in a cohort of patients undergoing primary AUS implantation (13, 14). In their initial study of 56 patients, O’Connor et al. found that in a matched analysis, double cuff placement was associated with a significantly greater rate of complete continence (p=0.008) and improvement in IIQ-7 scores (p=0.03) compared to single cuff placement (14). However, with longer follow-up (n=47), an average of 74.1 months for single cuff and 58 months for double-cuff, there was no significant difference in overall continence or quality of life between the two management strategies (13). We found similar results, with no evidence to support a significant difference in overall device survival or device infection/urethral erosion rates between single and double/tandem cuff placement in patients undergoing AUS revision for urethral atrophy.

Given these results, we have modified our practice and now, the typical initial approach to patients with recurrent bothersome stress urinary incontinence secondary to urethral atrophy is downsizing the urethral cuff, with tandem cuff reserved for cases of recurrent atrophy or when downsizing cannot be performed. An advantage to initial urethral cuff downsizing is that no additional periurethral dissection is necessary. However, it is worth noting that we found no difference in device infection/urethral erosion rates in our series between single and double cuff surgeries.

In cases where the smallest available cuff is already in-situ or cuff downsizing has previously failed, we proceed with additional urethral dissection and either moving the urethral cuff or placement of tandem urethral cuffs. As mentioned, all patients in this series underwent primary AUS placement at our institution, and thus relocation proximally was not physically possible. In this setting, tandem urethral cuff placement attempts to avoid increasing the pressure on the atrophic urethra segment and instead distributes additional compression to a second area of the urethra (9). It is worth noting that in order to account for the urethra tapering distally, we have used a transcorporal technique at times for added tissue bulk in the setting of revision for urethral atrophy (12). In the current series tandem transcorporal cuff placement was associated with adverse device survival compared to non-transcorporal tandem cuff placement. Given the study design it is difficult to discern if this is due to underlying factors that prompted a transcorporal approach, or the surgical technique. Similar to the experience of others (6, 15), we do not increase the pressure in the abdominal fluid reservoir in cases of urethral atrophy. Likewise, it is our preference to avoid downsizing to a 3.5cm urethral cuff, as there is little experience with this reported in the literature.

The limitations of our study, including its retrospective, non-randomized design should be noted. Given this, patient follow-up was not standardized and heterogeneous. While we verify follow-up through patient correspondence, some patients may undergo additional procedures with their local providers and may not be captured in our dataset. Additionally, we do not have functional outcomes available for patients that underwent revision for urethral atrophy, which limits our ability to determine if patient quality of life is different between these cohorts. As such, additional studies regarding the management of urethral atrophy following AUS placement, are needed to help define the optimal management strategy for these patients.

There was no significant difference in overall device survival in patients undergoing single cuff downsizing or tandem cuff placement during AUS revision for urethral atrophy. As such, we prefer to downsize the urethral cuff in the initial revision surgery to allow for tandem cuff placement during future revisions if needed.
